# Association between sarcopenia and levels of growth hormone and insulin-like growth factor-1 in the elderly

**DOI:** 10.1186/s12891-020-03236-y

**Published:** 2020-04-07

**Authors:** Ailin Bian, Yue Ma, Xinzi Zhou, Ying Guo, Wenyi Wang, Yiran Zhang, Xiaofei Wang

**Affiliations:** Geriatrics Department, Tianjin First Central Hospital, No. 24, Fukang Road, Nankai District, Tianjin, 300192 China

**Keywords:** Sarcopenia, Elderly, Growth hormone, Insulin-like growth factor-1, Mechanical growth factor

## Abstract

**Background:**

Age-related sarcopenia is a serious global health issue in elderly individuals and for the community as it induces disability and significant economic burden. The purpose of the study is to understand the factors associated with sarcopenia and the role of growth hormone (GH) and insulin-like growth factor (IGF-1) in the occurrence of sarcopenia.

**Methods:**

Elderly patients (*n* = 3276) were included in this cross-sectional study. Survey and measurement of body composition (bioelectrical impedance), grip strength, and step speed were performed according to the Asian Working Group on Sarcopenia (AWGS) diagnostic criteria. Hematological and hormonal indicators were compared between patients with and without sarcopenia in order to identify the associated factors.

**Results:**

There were significant differences in the demographic parameters between the sarcopenia and non-sarcopenia groups (all *P* < 0.05). There were significant differences between the two groups regarding the blood levels of GH, IGF-1, testosterone (T), and mechanical growth factor (MGF) (all *P* < 0.001). Correlation analyses showed that the appendicular skeletal muscle mass (ASMI) was positively associated with gender and BMI, and with GH, T, IGF-1, MGF, BUN, Cr, and Hb levels, but negatively associated with HDL-C (all *P* < 0.05). Logistic multivariable regression analysis showed that IGF-1, MGF, BMI, and gender were independently associated with appendicular skeletal muscle mass (ASMI) (all *P* < 0.05).

**Conclusions:**

GH and IGF-1 are associated with sarcopenia in the elderly. IGF-1 and MGF are independently associated with the reduction of skeletal muscle mass, along with BMI and gender.

## Background

Sarcopenia is an important global public health issue in the elderly due to the serious physical and psychological problems induced by this disease. Age-related sarcopenia is highly recognized in industrialized countries, since it causes a significant economic burden on health care services [1]. The Third National Health and Nutrition Examination Survey showed that the estimated medical expenses related to sarcopenia in the United States were $18.5 billion in 2000, accounting for 1.5% of the total medical expenses [2]. The U.S. Centers for Disease Control and Prevention (CDC) predicted that there were approximately 34 million people over the age of 65 in the United States in 2000, accounting for 13% of the total population and that this would increase to 70 million by 2030, accounting for 20% of the total population. In addition, 1.5 million elderly people over the age of 65 in the United States were admitted in medical institutions in 2006, and 33% of them were hospitalized due to inability of performing daily activities [3] indicating that the medical expenses associated with sarcopenia would further increase in the future.

Sarcopenia affects all elderly individuals, without any discrimination regarding race, gender, or wealth [4]. In Asia, the prevalence of sarcopenia is estimated to 4.1–11.5% in the general elderly population according to the Asian Working Group on Sarcopenia (AWGS) criteria, and the prevalence is higher if the European Working Group’s diagnostic criteria are used [5]. Weak elderly people often need assistance to perform their daily routine activities, due to the considerable loss of muscle mass and strength, and they often suffer serious injuries due to falls and fractures [4]. Losing the ability to live independently often induces psychological disorders, which may turn out to be painful not only for the individuals but also for their family members and caregivers.

Nevertheless, the pathogenesis and mechanisms of sarcopenia are still poorly understood. After the age of 60 years, a variety of hormones that promote the growth of muscle cells, such as testosterone (T), growth hormone (GH), insulin-like growth factor − 1 (IGF-1), and mechanical growth factor (MGF) are decreased [6]. In addition, increasing evidence suggests that the age-related effects of GH, IGF-1, androgen, and estrogen are associated with the incidence and pathogenesis of sarcopenia [4, 7]. The human skeletal muscle IGF-1 gene can be cleaved to produce three IGF-1 subtypes, IGF-1Ea, IGF-1Eb, and IGF-1Ec (also known as MGF). Muscle damage can induce the expression of the IGF-1 isoform MGF, followed by the appearance of calcium-dependent cell adhesion molecules and satellite cell marker, mucin. Since the peak of the IGF-1Ea expression level is attained after that of MGF, MGF may be the initial IGF-1 phenotype that triggers the activation of muscle satellite cells (SCs). Subsequently, the expression of IGF-1Ea is maintained during protein synthesis. Therefore, muscle repair is caused by the release of MGF after injury, followed by activation of SCs, and the level of SC reserve determines the potential of muscle regeneration [8, 9]. In addition, MGF is a major factor that activates skeletal muscle SCs to initiate skeletal muscle cell repair and promote their proliferation, when skeletal muscle cells are stimulated or damaged by external factors. With increasing age, the compliance of the body’s skeletal muscle may progressively reduce, and this decrease in compliance is accompanied by a decrease in the ability to synthesize and secrete MGF. Accordingly, studies have shown that sarcopenia is associated with decreased IGF-1 signaling, especially the IGF-1 gene-splicing isoform MGF, and the expression of MGF in skeletal muscle cells is decreased in patients with sarcopenia [10, 11].

Although it had been reported that supplementation of GH could increase the body mass of the elderly, the high incidence of adverse reactions and the high cost limit the applicability of this therapy [12]. The evaluation of alternative methods for improving the GH/IGF-1 axis in the elderly is currently underway. Testicular hormone replacement therapy can increase the muscle mass and strength and eventually reduce body fat in elderly men [7], but long-term randomized controlled trials are required to better understand the risk-benefit ratio of this therapy. Therefore, the evidence of age-related hormonal changes in the development of sarcopenia and the clinical utility of hormone supplementation in its management needs confirmation. At present, related studies in the Chinese population are rare.

This study aimed to evaluate the GH/IGF-1 axis in the pathogenesis of sarcopenia and to identify factors associated with it. The results could allow recommendations for comprehensive treatment and a global health management strategy to fight the problems associated with this disease.

## Methods

This cross-sectional study was conducted at the Tianjin First Central Hospital from December 2014 to August 2016. The study was approved by the local ethics committee. Written informed consent was obtained from all participants.

### Patient and public involvement

The study design was motivated by the elderly patients having to cope with sarcopenia and the subsequent lower quality of life. The patients were not involved in the design of this study. Except for their participation in the study itself, the patients were not otherwise involved in how the study was conducted. If they desired, the patients left their contact information, and they will receive a layman version of the results once they are published.

### Study participants

Elderly participants (≥60 years) who were admitted for consultation or health examination at the outpatient clinic were enrolled. The exclusion criteria were: 1) implanted metal objects such as cardiac pacemakers and fixed steel nails and others; 2) completely bed-ridden; 3) major physical disabilities; 4) permanently loss of activities of daily living (ADL); or 5) extracellular water (ECW)/total body water (TBW) value ≥0.40.

### Diagnostic criteria

According to the diagnostic criteria of sarcopenia from the AWGS, the patients who met criterion #1 and at least one of items #2 and #3 were diagnosed with sarcopenia and included: 1) muscle mass < 7.0 kg/m^2^ (male) or < 5.7 kg/m^2^ (female); 2) grip strength < 26 kg (male) or 18 kg (female); and 3) 4-m step speed < 0.8 m/s.^6^

### Questionnaire survey

The basic demographic characteristics of the participants were investigated using a self-made questionnaire. The main contents included 1) sex and age, 2) marital status (married/not married), 3) degree of education (primary/secondary/college or above); 4) profession (manual worker/office worker), 5) dietary habits (vegetarian/protein-rich diet), 6) dwelling condition (alone/with family), 7) smoking (yes/no), 8) drinking (yes/no), 9) sport habits (walking and Tai Chi/cycling and brisk walking/running and swimming/not exercising; and time < 60/60–150/> 150 min/week), and 10) disease history (hypertension, coronary heart disease, diabetes, cerebrovascular disease, and others). Exercise intensity was synthetically determined according to the mode and time of exercise: 1) low intensity: walking, Tai Chi, or the exercise time was < 60 min/week; 2) medium intensity: bicycle, quick walking, or the exercise time was 60–150 min/week, 3) medium and high intensity: running, swimming, or the exercise time was > 150 min/week. If there was almost no exercise or the time of low-intensity exercise was < 60 min/w, it was considered as no exercise. The researchers who participated in the survey (two graduate students and two nurses) have been trained before the investigation. They all used the same administration protocol.

### Measurement of body composition

Body composition was measured using the bioelectrical impedance method (BIA) (Human Body Composition Analyzer, model: INbody720, Korea). The participants wore light clothes and stood barefoot on the instrument. Body mass and height were measured, which were accurate to 0.1 kg and 0.1 cm, respectively. Body mass index (BMI) was calculated. The percentage of body fat (PBF), extracellular water (ECW), viscera fat area (VFA), arm muscle circumference (AMC), basal metabolic rate (BMR), bone mineral content (BMC), body cell mass (BCM), skeletal muscle mass (SMM), and other indicators were recorded. The appendicular skeletal muscle mass (ASMI) was reflected by the mass of the four limbs (kg)/height^2^ (m^2^).^6^

### Measurement of muscle strength and muscle function

Muscle strength was measured by grip strength using a grip force measuring instrument (WCS-II type, Beijing Zhuochuan Electronic Technology Co., Ltd.). Each hand was measured twice, and the highest value was taken. The muscle function was measured by 4-m step speed. The step speed test was the time required for a participant to walk 4 m at a usual speed from the standing position. It was measured using an electronic timer. The speed was calculated, and the cut-off level was ≤0.8 m/s.

### Blood test indicators

The participants fasted for 8 h. At 7:00–9:00 am, blood was taken through vein puncture in a vacuum blood collection tube and allowed to sit for 30 min. The serum was separated at 4 °C. The indicators included hemoglobin (Hb), total cholesterol (TC), triacylglycerol (TG), high-density lipoprotein cholesterol (HDL-C), low-density lipoprotein cholesterol (LDL-C), serum albumin (ALB), urea nitrogen (BUN), and creatinine (Cr). All indicators were measured on the same day. Aliquots of 400 μl of serum were prepared in two Eppendorf tubes and frozen at − 70 °C. Those indicators are routine markers for determining the health condition of elderly individuals.

Hb was detected by the sodium dodecyl sulfonate method, whereas ALB, TG, and UREA/BUN were detected by colorimetric methods. TC, HDL-C, and LDL-C were measured by enzymatic colorimetric methods.

### Measurement of hormones and cytokines

The serum stored at − 70 °C was used to measure the levels of GH, IGF-1, T, and MGF in batches and using ELISA (Santa Cruz Biotechnology, Santa Cruz, CA, USA). The minimum detection limits provided by the manufacturer are: 0.1 ng/mL for GH, 1.0 ng/mL for IGF-1, 0.1 ng/mL for T, and 1.0 pg/mL for MGF. The intra-plate and inter-batch differences were all ≤6%. All samples were assayed in duplicates, and the average value was taken. The correlation coefficient of the linear regression curve was obtained by fitting the concentration of the standard sample and its measured absorbance value (all r ≥ 0.98).

### Statistical analysis

Statistical analysis was performed using SPSS 20.0 (IBM, Armonk, NY, USA). Continuous data are expressed as means ± standard deviation and were analyzed using the independent sample t-test. Categorical data are presented as frequencies and were analyzed using the chi-square test. The Pearson correlation analysis was used for the continuous variables. The Spearman correlation analysis was performed for the ordinal variables. The interactions between multiple factors were analyzed by binary logistic regression and multiple linear regression. *P* values < 0.05 were considered statistically significant.

## Results

A total of 3276 elderly participants, including 2325 males and 951 females, were enrolled in this study. The participants were 60–87 years of age (mean of 74 ± 18 years).

### The frequency of sarcopenia

According to the diagnostic criteria of the AWGS for sarcopenia, the frequency of sarcopenia in the study population was 12.82% (420/3276), or 11.10% (258/2325) and 17.03% (162/951) in elderly men and women, respectively. The frequency of sarcopenia in women aged 60–69 and 70–79 years was higher than that in men of the corresponding age (all *P* < 0.05). There was no significant difference in the frequency of sarcopenia between men and women aged 80–87 years (*P* > 0.05). The frequency of sarcopenia increased with age (*P* < 0.05) (Table 1).

**Table 1:** Comparison of the frequency of sarcopenia among adults of different genders in Tianjin region under the Asian diagnostic criteria

Age	Total (n)	Male		Female		χ^2^ value	*P* value
		n	Morbidity	n	Morbidity		
60–69	1137	726	33 (4.54)	411	42 (10.22)	11.847	0.001
70–79	1209	903	87 (9.63)	306	60 (19.61)	15.992	< 0.001
80–87	930	696	138 (19.82)	234	60 (25.64)	2.24	0.135
χ^2^_tendency_value		62.54		26.776			
*P* value		< 0.001		< 0.001			

### Clinical features and factors associated with sarcopenia

All participants were divided into the sarcopenia and non-sarcopenia groups. There were significant differences between the two groups in age, gender, BMI, smoking and drinking history, disease history, and exercise habits, as well as in body composition indices such as VFA, BMR, BCM, BMC, and AMC (all *P* < 0.05) (Table 2). Patients with sarcopenia were older, had a long history of smoking and drinking, more disease history, fewer exercises, and lower BMI, visceral fat area, bone mineral content, body cell mass, and basal metabolic rate compared with those in the non-sarcopenia group. The multivariable analysis suggested that gender, smoking history, exercise habits, BMI, VFA, and BCM were associated with sarcopenia (all *P* < 0.05) (Table 3).

**Table 2:** Comparison of demographic and basic clinical characteristics between the sarcopenia group and non-sarcopenia group

Parameters	Sarcopenia group	Non-sarcopenia group	*P*
	*n* = 420	*n* = 2856	
Male/female	258/162	2067/789	0.001
Age	77.71 ± 8.437	71.26 ± 7.529	< 0.001
BMI	21.125 ± 2.313	24.855 ± 2.841	< 0.001
Smoking history	13 (3.2%)	327 (11.4%)	0.002
Drinking history	13 (3.1%)	232 (8.15)	0.004
Number of diseases ≥3	81 (19.3%)	129 (4.5%)	< 0.001
Low protein diet	81 (19.3)	534 (18.6)	0.343
Exercise habit (No)	121 (28.8%)	267 (9.3%)	< 0.001
PBF (%)	27.127 ± 7.160	26.593 ± 7.296	0.639
BMC (kg)	2.331 ± 0.306	2.932 ± 0.407	< 0.001
AMC (cm)	22.279 ± 1.469	26.005 ± 4.678	< 0.001
VFA (cm^2^)	72.854 ± 23.907	86.745 ± 31.215	0.004
BMR (kcal)	1248.85 ± 127.699	1503.08 ± 156.320	< 0.001
BCM (kg)	26.069 ± 3.803	33.903 ± 4.796	< 0.001
FFM of Left Arm (kg)	1.957 ± 0.409	2.888 ± 0.895	< 0.001
FFM of Right Arm (kg)	2.007 ± 0.439	2.952 ± 1.026	< 0.001
FFM of Right Leg (kg)	6.358 ± 1.278	8.360 ± 1.357	< 0.001
FFM of Left Leg (kg)	6.313 ± 1.274	8.290 ± 1.319	< 0.001
ASMI (kg/m^2^)	6.17 ± 0.721	7.75 ± 1.087	< 0.001
Grip strength (kg)	22.358 ± 6.773	29.411 ± 8.116	< 0.001
Hb (g/L)	135.23 ± 11.08	137.70 ± 12.69	0.338
ALB (g/L)	42.68 ± 2.13	43.17 ± 1.82	0.248
BUN (mmol/L)	5.49 ± 1.53	5.86 ± 1.61	0.269
Cr (μmol/L)	73.57 ± 15.38	76.89 ± 17.36	0.347
LDL (mmol/L)	3.11 ± 0.78	3.24 ± 0.81	0.407
HDL (mmol/L)	1.38 ± 0.31	1.31 ± 0.33	0.201

History of the disease included diabetes, hypertension, coronary heart disease, cerebral infarction, and others. A low-protein diet referred to a diet mainly contained carbohydrates and vegetables. No exercise habit meant no physical activity or exercise time < 1 h/week

*Abbreviation*: *BMI* body mass index, *PBF* percentage of body fat, *VFA* viscera fat area, *AMC* arm muscle circumference, *BMR* basal metabolic rate, *BMC* bone mineral content, *BCM* body cell mass, *Hb* hemoglobin, *TC* total cholesterol, *TG* triacylglycerol, *HDL-C* high-density lipoprotein cholesterol, *LDL-C* low-density lipoprotein cholesterol, *ALB* serum albumin, *BUN* urea nitrogen, *Cr* creatinine, *ASMI* appendicular skeletal muscle mass, *FFM* fat-free mass

**Table 3:** Analysis of risk factors for sarcopenia, multivariable logistic regression analysis, and the method of variable inclusion was the enter method

Variables	*P* value	OR	95% CI
Gender	< 0.001	5.058	3.057–19.852
Age	0.342	0.925	0.788–1.086
BMI	0.005	0.104	0.022–0.498
BMC	0.078	1.05	0.2–1.14
AMC	0.271	0.264	0.025–2.828
VFA	0.009	1.162	1.038–1.300
BMR	0.100	0.750	0.603–0.932
BCM	0.038	5.345	1.429–19.994
Grip strength	0.022	0.834	0.714–0.974
Drinking history	0.298	4.746	0.253–8.903
Smoking history	0.042	1.897	0.163–2.069
History of diseases	0.703	0.381	0.085–1.126
Exercise habit	0.012	1.109	0.171–1.201

*Abbreviations*: *BMI* body mass index, *BMC* bone mineral content, *AMC* arm muscle circumference, *VFA* viscera fat area, *BMR* basal metabolic rate, *BCM* body cell mass

### Correlation analysis between skeletal muscle mass and hormones

**Fig. 1:**
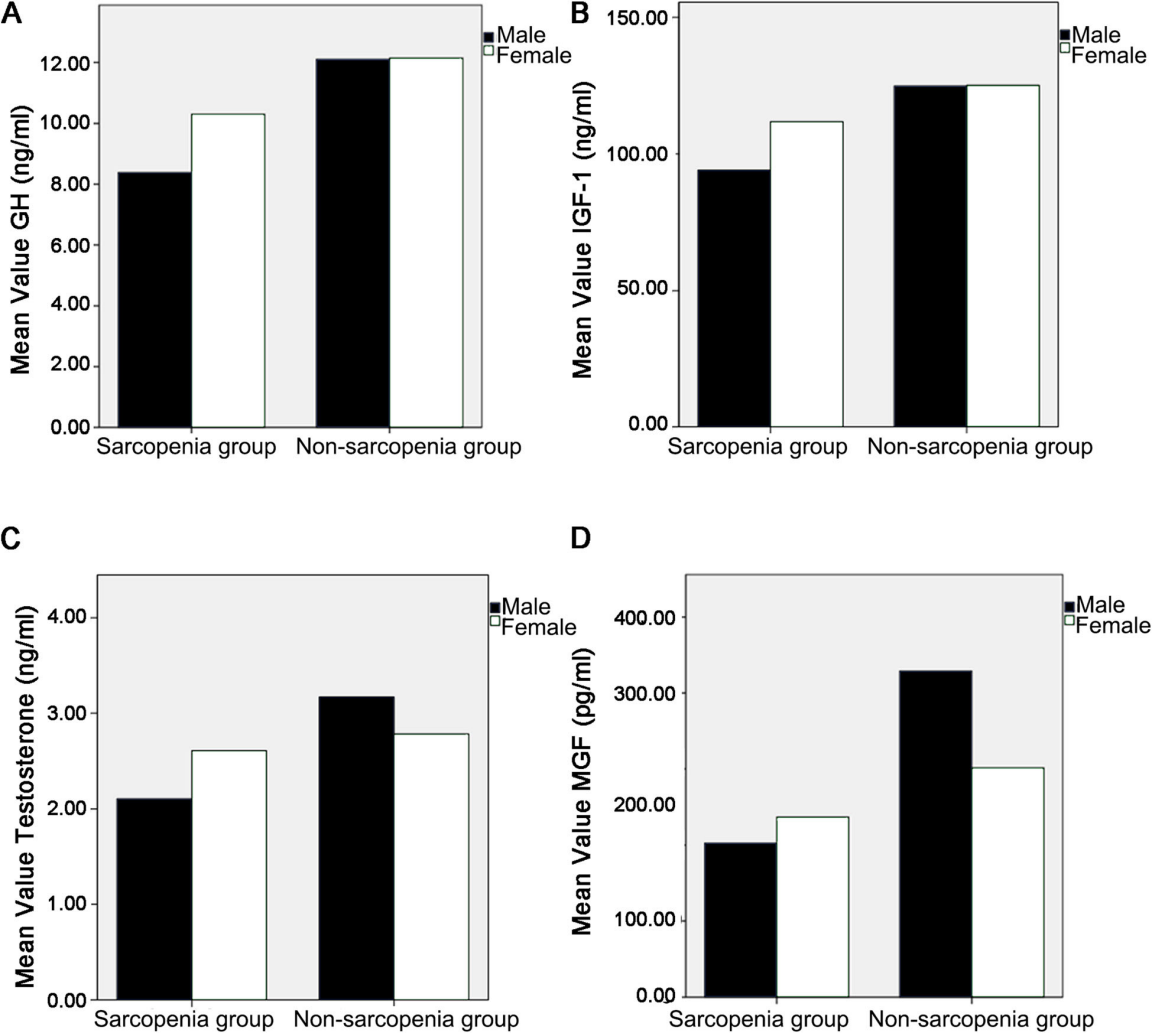
Hormone levels in participants with and without sarcopenia and according to sex. **a** Growth hormone (GH). **b** Insulin-like growth factor (IGF)-1. **c** Testosterone (T). **d** Mechanical growth factor (MGF). All *P* < 0.05 for sarcopenia vs. non-sarcopenia

The hormonal analysis showed that GH (9.18 ± 2.36 vs. 12.20 ± 3.93 ng/mL), IGF-1 (98.53 ± 28.45 vs. 136.41 ± 48.95 ng/mL), T (2.20 ± 0.77 vs. 3.14 ± 1.23 ng/mL), and MGF (210.84 ± 67.01 vs. 392.98 ± 226.34 pg/mL) were lower in the sarcopenia group compared with the non-sarcopenia group (all *P* < 0.001) (Fig. 1). According to the correlation analysis, ASMI was positively correlated with GH, T, IGF-1, and MGF levels; ASMI was also positively associated with gender, BMI, plasma BUN, Cr, and Hb levels, and negatively with plasma HDL-C levels (all *P* < 0.05) (Table 4). The results also showed that IGF-1 was positively correlated with GH, T, MGF, BMI, BMC, and Hb, and negatively with age (all *P* < 0.05).

**Table 4:** Correlation between skeletal muscle mass and hormones

Items	r	*P*
GH	0.217	0.042
T	0.272	0.010
IGF-1	0.245	0.021
MGF	0.335	0.001
Bun	0.253	0.017
Cr	0.483	0.001
HDL-C	−0.272	0.010
Hb	0.353	0.001
BMI	0.552	0.001
Gender	0.672	0.001

*Abbreviations*: *GH* growth hormone, *T* testosterone, *IGF-1* insulin-like growth factor, *MGF* mechanical growth factor, *BUN* blood urea nitrogen, *Cr* creatinine, *HDL-C* high density lipoprotein cholesterol, *Hb* hemoglobin, *BMI* body mass index

### IGF-1 and MGF are independently associated with ASMI

The multivariable regression analysis was performed using ASMI as the dependent variable, and gender, BMI, GH, T, IGF-1, MGF, BUN, Cr, HDL-C, Hb as independent variables. The results showed that IGF-1, MGF, BMI, and gender were independently associated with ASMI (all *P* < 0.05) (Table 5).

**Table 5:** Multivariable linear regression analysis using the ASMI as dependent and other factors as independent variables

Factors	*β*	*Std*	*t*	*P*
GH	− 0.017	0.024	−0.695	0.489
T	−0.011	0.072	−0.155	0.877
IGF-1	0.004	0.002	2.677	0.009
MGF	0.001	0	2.352	0.021
Bun	0.006	0.042	0.134	0.894
Cr	0.004	0.005	0.808	0.422
HDL-C	0.205	0.184	1.113	0.269
Hb	−0.003	0.005	−0.683	0.496
BMI	0.152	0.02	7.516	< 0.001
Gender	1.298	0.142	9.173	< 0.001

*Abbreviations*: *GH* growth hormone, *T* testosterone, *IGF-1* insulin-like growth factor, *MGF* mechanical growth factor, *BUN* blood urea nitrogen, *Cr* creatinine, *HDL-C* high density lipoprotein cholesterol, *Hb* hemoglobin, *BMI* body mass index

## Discussion

Age-related sarcopenia is a serious global health issue in elderly individuals and for the community as it induces disability and significant economic burden. The purpose of the study was to understand the factors associated with sarcopenia and the role of GH and IGF-1 in the occurrence of sarcopenia. The results suggest that GH and IGF-1 are associated with sarcopenia in the elderly. IGF-1 and MGF are independently associated with the reduction of skeletal muscle mass, along with BMI and sex.

Sarcopenia is an age-related disease, and its morbidity increases significantly with age. This study suggested that there are age and gender differences in the occurrence of sarcopenia. As the age increased, the frequency also increased, especially in participants > 80 years of age. The frequency of sarcopenia in women aged 60–79 was higher than in men (same age groups), but there was no gender difference in the frequency over the age of 80. In addition to age, there were many other factors associated with sarcopenia, including lifestyle, exercise habits, disease status, and hormone levels. The results suggest that smoking history and less exercise habits were associated with sarcopenia, suggesting that unhealthy lifestyles such as smoking and less exercise may be associated with the occurrence of sarcopenia, which was consistent with previous studies [13]. The coexistence of unhealthy lifestyles and various bad habits had been reported to be associated with sarcopenia [14–16]. Robinson et al. reported that risk factors for adverse lifestyles (including smoking, obesity, poor diet, and low physical activity) were strongly, inversely, and hierarchically linked to body functions of males and females. During the observation of relationship between the cumulative effects of adult health behaviors and sarcopenia, the Whitehall II study, after 5, 10, and 17 years of evaluation, found that all people who had unhealthy behaviors at middle-aged periods (including smoking, intemperance drinking, low intake of fruits and vegetables, and physical inactivity) had low walking speeds after 17 years [14]. In short, the coexistence and extended duration of unhealthy behaviors, especially diet and lack of physical activity, were associated with sarcopenia and, in particular, it had a far-reaching influence on body functions. Encouraging healthy lifestyles in daily life may have the potential to improve the body functions of elderly people.

BMI and sex were independently associated with ASMI. BMI and sex are independent risk factors for sarcopenia and have been confirmed in other studies. Indeed, a study of 1971 community-dwelling older adults in Kashiwa City (Japan) found that 14.2% of men and 22.1% of women were sarcopenic [17]. Another survey in Japan reported prevalence rates of sarcopenia of 21.8% in men and 22.1% in women, which were age- and sex-dependent [18]. In addition, male participants younger than 75 were significantly less sarcopenic than women of the same age group (65–69 years: 2.8% vs. 11.3%; 70–74 years: 5.3% vs. 11.8%). In a study of Japanese women aged above 75 years, the prevalence rates of pre-sarcopenia, sarcopenia, and severe sarcopenia were 23.8, 11.2, and 4.6%, respectively [19]. Older age, lower BMI, history of heart disease, and hyperlipidemia were all related to the development and progression of sarcopenia [19].

A cross-sectional study by Szulc et al. found that smokers had lower skeletal muscle mass index (− 3.2%) compared with those who never smoked [20]. In elderly people, there was a strong correlation between smoking and muscle strength reduction and physical ability decline, such as grip strength, chair ascending speed, tug/walking speed, balance ability, etc. [21, 22]. A longitudinal study of healthy young adults showed that smoking was negatively correlated with knee muscle strength after adjusting for other lifestyle factors [23]. Nevertheless, there are also reports demonstrating that smoking is not an important risk factor for low muscle mass in a fully adjusted model [24]. Moreover, a recent MET analysis also indicated that smoking might have little effect on the occurrence and development of sarcopenia [25]. Therefore, the relationship between smoking and sarcopenia remains to be further explored.

The results of this study also suggested that the coexistence of multiple diseases was associated with the occurrence of sarcopenia, which was consistent with previous studies [26, 27]. The presence of many chronic diseases such as chronic obstructive pulmonary disease (COPD), cardiovascular diseases, and cancer had been shown to be associated with loss of muscle mass [26]. Studies in Taiwan (The Sarcopenia and Translational Aging Research in Taiwan) showed that comorbidities were associated with lower grip strength and physical activity, walking speed, and tug (TUG). Particularly, these associations were stronger in elderly people with low muscle mass. After adjusting for confounding factors, participants with two or more chronic diseases and low muscle groups were weaker than those without any risk factors [27].

In addition to the impact of lifestyle on skeletal muscle mass and function, the present study showed that GH/IGF-1 levels were significantly different between the sarcopenia and non-sarcopenia groups. Skeletal muscle mass was significantly and positively correlated with GH, T, IGF-1, and MGF, and the results of the multivariable regression analysis eventually showed that IGF-1 and MGF were independently associated with skeletal muscle loss. IGF-1 and MGF levels had a stronger influence on skeletal muscle mass, which probably played vital roles in the occurrence of sarcopenia.

IGF-1 is mainly synthesized (75%) by the liver and secreted into the blood, and its synthesis and secretion are mainly controlled by GH. IGF-1 is also synthesized in skeletal muscles and regulates muscle growth in an autocrine/paracrine manner. IGF-1 exerts its effects by activating the IGF-1 receptor, which is widely distributed and allows blood-transported IGF-1 to coordinate growth balance in multiple tissues and organs. In comparison, autocrine/paracrine IGF-1 stimulates imbalanced local growth, which is independent of systemic GH [28, 29]. Recent studies had shown that IGF-I is involved in increasing muscle mass and strength, reducing degeneration, inhibiting prolonged and excessive inflammatory processes due to toxin damage, and increasing the proliferative potential of satellite cells [30]. IGF-I is currently considered as a biomarker of health, and the high levels of circulating concentrations are positively correlated with many body composition and cardiovascular health-related parameters, but negatively correlated with body fat. IGF-I is also positively associated with aerobics and muscle endurance measurements [31].

We further analyzed the relationship between hormone levels and other factors and found that IGF-1 was significantly correlated with age, BMI, Hb, and BMC. These findings indicated that in addition to age, IGF-1 was also related to the nutritional status of the body, which is consistent with some previous studies [32, 33]. Nutritional status is an important factor affecting the plasma IGF-1 levels, shown by the fact that fasting for 7 days can induce a 50% reduction of the plasma IGF-1 levels [32, 33]. In order to maintain normal plasma IGF-1 levels, at least 20 kCal/kg and 0.6 g/kg protein must be ingested daily. Pathological conditions such as malnutrition, severe disease, sepsis, high-dose exogenous glucocorticoid use, and inflammation are significantly associated with low levels of IGF-1 mRNA in the muscle [34].

After the age of 60 years, a variety of hormones that promote the growth of muscle cells, such as T, GH, and IGF-1 are decreased [6]. High levels of GH/IGF-1 are necessary for growth and development during adolescence. Plasma IGF-1 levels are low at birth, rising to a peak in adolescence, up to sevenfold, and was 40–50% of the peak in adolescence by the age of 20. IGF-1 levels decrease by 50% by the age of 60. This change is partly due to age-dependent changes in GH secretion [5, 35–37]. Changes in the GH/IGF-1 level cause a decrease in the levels of protein anabolism in skeletal muscle cells, which ultimately leads to changes in the structure and function of skeletal muscle cells. Therefore, the reduction of GH/IGF-1 plays a key role in the loss of skeletal muscle mass.

Collectively, these findings may suggest that it could be possible, theoretically, to increase skeletal muscle mass by the administration of IGF-1 or MGF. Nevertheless, the dose problems and potential side effects are still the major obstacles in its clinical application. Except for the ALS study [38, 39], few phase III human trials have been conducted. In fact, many drug delivery strategies did not achieve much success so far, most likely because local concentrations of IGF-1 could not be maintained. In order to avoid the potential carcinogenic effects associated with IGF-1, there were currently explorations of the therapeutic potential of mgf-24a-e peptides. Since the monopeptide itself has the ability to significantly enhance SC activation, proliferation, and fusion, it could be used for muscle repair and maintenance [38]. Using the synthetic mgf-24a-e peptide succeeding to treat primary human muscle cell culture and improve the proliferative ability of myoblasts isolated from neonatal and young adult muscles [13, 40]. Nevertheless, whether this peptide can increase the proliferation of myoblasts isolated from the muscles of the elderly people has not been confirmed. T replacement therapy can increase muscle mass and strength, and reduce body fat in elderly men, but long-term randomized controlled trials are still needed to better estimate the risk-benefit ratio of these therapies before they can be clinically recommended.

The prevalence of sarcopenia in senior citizens > 80 years old was slightly higher in females than in males (25.6% vs. 19.8%), but without significant difference. There were no gender differences in GH, IGF-1, T, and MGF between males and females (all *P* > 0.05) (Supplementary Table S1). The proportion of elderly men taking part in sports and having certain exercise intensity was higher than that of elderly women (37.5% vs. 22%), but the proportion of elderly men smoking and drinking was higher than in elderly women (6.3 and 3.87% vs. 0%). Therefore, those results might explain the weakening of the sex differences in the prevalence of sarcopenia in older adults. At the same time, it should be pointed out that most of the cases collected in this study were from a highly educated population. Moreover, the number of cases in this age group was small, which could not truly reflect the situation of the population.

Our study has some limitations, as it was limited to a single center with a restricted population. In addition, because of the lack of follow-up, we could not derive valuable information on the relationship between GH and IGF-1 and its long-term effects on sarcopenia patients.

## Conclusions

In conclusion, many factors (including age, gender, lifestyle, diet, and exercise) play roles in the development of sarcopenia in elderly people. GH/IGF-1 levels play an essential role in maintaining skeletal muscle quality. Particularly, IGF-1 and MGF were independently associated with skeletal muscle mass. Clinical trials are necessary before implementing IGF-1 or MGF therapy in clinical practice to maintain skeletal muscle mass and function in the elderly and to improve their quality of life.

## Supplementary information

**Additional file 1: Table S1.** Subgroup analysis of the sarcopenic group >80 years of age.

## Data Availability

The datasets used and analyzed during the current study are available from the corresponding author on reasonable request.

## Abbreviations

GH: Growth hormone
IGF-1: Insulin-like growth factor-1
T: Testosterone
MGF: Mechanical growth factor
ASMI: Appendicular skeletal muscle mass
CDC: Centers for Disease Control and Prevention
BIA: Bioelectrical impedance analysis
PBF: Percentage of body fat
ECW: Extracellular water
VFA: Viscera fat area
AMC: Arm muscle circumference
BMR: Basal metabolic rate
BMC: Bone mineral content
BCM: Body cell mass
SMM: Skeletal muscle mass
