# Supplementary material for: Association between sarcopenia and levels of growth hormone and insulin-like growth factor-1 in the elderly
Source: BMC Musculoskelet Disord. 2020 Apr 7;21:214. doi: 10.1186/s12891-020-03236-y (PMC7140321; doi:10.1186/s12891-020-03236-y)
Supplement: Supplementary file 1 — Additional file 1: Table S1. Subgroup analysis of the sarcopenic group >80 years of age. [file 12891_2020_3236_MOESM1_ESM.docx]

**Supplementary Table S1.** Subgroup analysis of the sarcopenic group >80 years of age.

| Parameters | Male (n=80) | Female (n=35) | *t* | *P* |
| --- | --- | --- | --- | --- |
| BMI | 22.89±2.88 | 22.36±2.41 | -1.38 | 0.19 |
| PBF (%) | 25.42±4.99 | 29.69±8.07 | 1.56 | 0.13 |
| BMC (kg) | 2.68±0.32 | 2.14±0.19 | -4.13 | <0.01 |
| AMC (cm) | 24.17±2.02 | 21.63±1.41 | -3.01 | 0.01 |
| VFA (cm^2^) | 79.24±22.74 | 83.71±22.32 | 0.44 | 0.67 |
| BMR (kcal) | 1407.25±110.79 | 1146.71±74.16 | -5.65 | <0.01 |
| BCM (kg) | 30.71±3.39 | 22.89±2.12 | -5.60 | <0.01 |
| SLM (kg) | 45.33±4.84 | 36.41±8.40 | -3.24 | <0.01 |
| ASMI (kg/m^2^) | 7.20±0.70 | 5.59±0.36 | -5.75 | <0.01 |
| Grip strength (kg) | 24.15±5.14 | 14.50±3.13 | -4.53 | <0.01 |
| Hb (g/L) | 141.44±17.34 | 131.14±11.73 | -1.43 | 0.17 |
| ALB (g/L) | 42.43±2.69 | 42.20±2.18 | -0.19 | 0.85 |
| BUN (mmol/L) | 6.94±1.70 | 5.45±1.57 | -2.05 | 0.06 |
| Cr (mmol/L) | 93.31±18.88 | 69.14±12.42 | -3.09 | 0.01 |
| HDL (mmol/L) | 1.22±0.35 | 1.30±0.15 | 0.61 | 0.55 |
| LDL (mmol/L) | 2.84±0.69 | 2.96±0.75 | 0.37 | 0.71 |
| GH | 10.40±4.37 | 8.70±1.25 | -1.00 | 0.33 |
| IGF1 | 104.45±32.85 | 99.20±24.18 | -0.38 | 0.71 |
| T | 2.81±1.67 | 1.92±0.28 | -1.38 | 0.18 |
| MGF | 307.46±217.70 | 221.21±99.40 | -0.99 | 0.33 |

Abbreviation: body mass index (BMI), percentage of body fat (PBF), viscera fat area (VFA), arm muscle circumference (AMC), basal metabolic rate (BMR), bone mineral content (BMC), body cell mass (BCM), hemoglobin (Hb), total cholesterol (TC), triacylglycerol (TG), high density lipoprotein cholesterol (HDL-C), low density lipoprotein cholesterol (LDL-C), serum albumin (ALB), urea nitrogen (BUN), creatinine (Cr), appendicular skeletal muscle mass (ASMI), skeletal muscle mass (SMM), growth hormone (GH), insulin-like growth factor-1 (IGF-1), testosterone (T), mechanical growth factor (MGF).
